# Supplementary material for: Rapid Recalibration of Peri-Personal Space: Psychophysical, Electrophysiological, and Neural Network Modeling Evidence
Source: Cereb Cortex. 2020 May 7;30(9):5088–106. doi: 10.1093/cercor/bhaa103 (PMC7391419; doi:10.1093/cercor/bhaa103)
Supplement: Noel_EXTENDED_DATA_bhaa103 [file noel_extended_data_bhaa103.docx]

EXTENDED DATA FOR:

Rapid Recalibration of Peri-Personal Space; Psychophysical, Electrophysiological, and Neural Network Modeling Evidence

Jean-Paul Noel^1, 2, 3, *^, Tommaso Bertoni^4^, Emily Terrebonne^2^, Elisa Pellencin^5^, Bruno Herbelin^6, 7^, Carissa Cascio^2, 8^, Olaf Blanke^6, 7^, Elisa Magosso^9^, Mark T. Wallace^2, 8, 10, 11^, Andrea Serino^4^

^1^ Neuroscience Graduate Program, Vanderbilt Brain Institute, Vanderbilt University Medical School, Vanderbilt University, Nashville, TN 37235, USA

^2^ Vanderbilt Brain Institute, Vanderbilt University Medical School, Vanderbilt University, Nashville, TN 37235, USA

^3^ Center for Neural Science, New York University, New York City, NY 10003, USA

^4^ MySpace Lab, Department of Clinical Neurosciences, University Hospital of Lausanne, University of Lausanne, Lausanne, Switzerland

^5^ Department of Psychology and Cognitive Science, University of Trento, Corso Bettini 84, 38068, Rovereto, Trento, Italy

^6^ Laboratory of Cognitive Neuroscience, Brain Mind Institute, Ecole Polytechnique Federale de Lausanne, Lausanne, Switzerland

^7^ Center for Neuroprosthetics, Campus BioTech, Geneva, Switzerland

^8^ Department of Psychiatry and Behavioral Sciences, Vanderbilt University Medial Center, Nashville, TN 37235, USA

^9^ Department of Electrical, Electronic, and Information Engineering "Guglielmo Marconi", University of Bologna, Cesena, Italy

^10^ Department of Hearing and Speech Sciences, Vanderbilt University Medical Center, Nashville, TN 37235, USA

^11^ Department of Psychology, Vanderbilt University, Nashville, TN 37235, USA

Corresponding Author:

Jean-Paul Noel

Center for Neural Science, NYU

4 Washington Pl, New York, NY 1003

Email: [jpn5@nyu.edu](mailto:jpn5@nyu.edu)

**Supplementary Figures**

**Figure S1. Rapid Recalibration of PPS, including all subjects.** Given that a larger number of participants were removed from analyses in the main text given poor fits, we repeated the analyses while including all participants and performing simple analyses of variance, as opposed to data fitting. As in the main text, these analyses demonstrated a general visuo-tactile multisensory facilitation with respect to tactile reaction time (dashed vs. solid lines: t = 6.71, p = 7.03x10^11^). Further, a one-way ANOVA demonstrated that visuo-tactile proximity played a role in further enhancing multisensory facilitation (F = 5.04, p = 0.02). Most importantly, a 2 (t-1 smaller vs. t-1 larger) x 4 (distances: D2-D5), demonstrated a significant interaction (F = 3.37, p = 0.04), confirming that trial history impacts PPS encoding.

**Figure S2. General Linear Model (GLM) predicting reaction times given n-back trials.** Results from fitting a GLM (up to 10 trials, no interaction between terms, canonical link function yielding a gamma distribution) suggest that trials t and t-1 directly impact reaction times at trial t. The impact of trial t-1 is approximately 26-27 % (27% in Experiment 1, 26% in Experiment 2) that of trial t (y-axis weight normalized the weight of trial t, x-axis, n-back). Results from Experiment 1 suggest that trial t-2 also significantly impacts reaction times at trial t (~15% that of trial t), but this was not supported by Experiment 2. Of note, this does not mean that trials before t-1 or t-2 do not impact reaction times, but likely that they do so via an accumulated effect requiring the interplay between multiple n-back trials.

**Figure S3. Global Field Power while subsampling trials to match across conditions.** The results are virtually identical to those in the main text, strongly suggesting that given the number of repetitions represented we had reached an asymptote in signal-to-noise ratios. While matching the number of V, T, and VT repetitions at the single subject level, we observer supra-additivity (p < 0.01) between 123-160ms and 204-226ms post-stimuli onset.

**Figure S4. The modulation of GFP response as a function of distance is specific to VT stimuli.** A 2 (V vs. VT) x 5 (distances) on the mean GFP between 130 and 150ms post-stimuli onset demonstrated a significant main effect of distance (F = 6.41, p = 6.15x10^-5^) and stimuli condition (F = 67.24, p = 1.11x10^-14^). Most importantly, there was a significant interaction between these variables (F = 28.79, p = 8.12x10^-20^) driven by the fact that while this peak was modulated by distance in VT presentations (main text), it was not in the case of visual-only stimulation (F = 1.17, p = 0.32).

**Figure S5. Rapid recalibration of PPS at different velocities.** Rapid recalibration is apparent at 75cm/s (the velocity used in the behavioral study here), but not when stimuli are faster (100cm/s) or slower (25cm/s or 50cm/s). At large velocities, the PPS expands (as demonstrated in Noel et al., 2018), and reaction times near the body show a floor effect. In turn, the effect of Hebbian learning is only apparent at far distances. At slow velocities (25cm/s and 50cm/s), we speculate that the intrinsic neural adaptation driven the remapping of PPS with velocity accounts for much of the neural response, and hence the impact of Hebbian learning is masked. This demonstrates a tight interplay between dynamic (neural adaptation accounting for re-sizing with different velocities; Noel et al., 2018) and plastic (Hebbian learning account for rapid recalibration) aspects of PPS.
